# Analyzing the Role of Physical Therapy in Recovery From Sport-Related Orthopedic Injuries

**DOI:** 10.7759/cureus.88613

**Published:** 2025-07-23

**Authors:** Shakir Ullah, Muhammad Adeel Zafar, Komal Saleem, Muhammad Tayyab, Kamran Khan, Atizaz A Jan, Haziq Dad Khan, Bilal Ahmad

**Affiliations:** 1 Orthopaedics, Gajju Khan Medical College, Bacha Khan Medical Complex, Swabi, Swabi, PAK; 2 Respiratory Medicine, NHS Wales UK, Bridgend, GBR; 3 Obstetrics and Gynaecology, University Hospital of Wales, Cardiff, GBR; 4 Trauma and Orthopaedics, Hayatabad Medical Complex Peshawar, Peshawar, PAK; 5 Trauma and Orthopaedics, Bradford Teaching Hospitals, Bradford, GBR; 6 Department of Orthopaedic Surgery, MTI Mardan Medical Complex, Mardan, Mardan, PAK; 7 Trauma and Orthopaedics, University Hospital Crosshouse, Kilmarnock, GBR; 8 Trauma and Orthopaedics, Saidu Group of Teaching Hospitals, Swat, Swat, PAK; 9 Orthopaedic Surgery, Bacha Khan Medical College, Mardan, Mardan, PAK; 10 Orthopaedic Surgery, Saidu Group of Teaching Hospitals, Swat, PAK; 11 Trauma and Orthopaedics, Queen Elizabeth Hospital Birmingham, Birmingham, GBR; 12 Trauma and Orthopaedics, Khyber Teaching Hospital, Peshawar, Peshawar, PAK

**Keywords:** functional recovery, musculoskeletal injuries, orthopedic injury, physical therapy, return to sports, sports rehabilitation

## Abstract

Background: Orthopedic injuries from sports are frequent in physically active people, and they often need organized therapy to restore function and stop recurrence.

Objective: To evaluate the role of physical therapy in functional recovery, performance improvement, and re-injury reduction among patients with sport-related orthopedic injuries.

Methodology: This descriptive observational study was conducted at the Department of Physical Therapy, Bacha Medical College Mardan, from March 2023 to February 2024. The study included 216 individuals with orthopedic injuries connected to sports who had undergone at least four weeks of physical therapy and were between the ages of 18 and 50. SPSS version 25 (IBM Corp., Armonk, NY, USA) was used to gather and analyze data on demographics, injury kind and location, therapeutic methods, and recovery results.

Results: Out of the 216 participants, 84 (38.89%) were female and 132 (61.11%) were male. The most common injuries were ligament sprains or tears (33.33%), muscle strains (22.69%), fractures (18.98%), joint dislocations (16.67%), and tendon injuries (8.33%). The knee was the most frequently affected site (30.09%), followed by the shoulder (22.69%) and ankle (20.37%). Therapeutic exercises were administered to 87.50% of patients, manual therapy to 72.22%, and electrotherapy to 62.04%. Significant improvements were observed: 86.11% reported pain reduction, 80.09% improved range of motion, and 73.61% achieved functional recovery. A total of 63.89% resumed athletic activities, while 8.80% experienced a re-injury.

Conclusion: Physical therapy interventions significantly support recovery, functional improvement, and safe return to sport in patients with orthopedic injuries.

## Introduction

Sport-related orthopedic injuries are a significant concern for athletes and physically active individuals, with potential adverse effects on their physical, psychological, and social well-being [1]. Globally, such injuries account for an estimated 20-40% of musculoskeletal clinic visits, particularly among those engaged in high-intensity sports, and are increasingly common due to rising participation in both amateur and professional athletics [2,3]. These injuries most often involve the lower and upper limbs, including sprains, strains, ligament tears, fractures, and joint dislocations [2]. They can impair an athlete’s performance, prolong recovery, and, in severe cases, jeopardize the ability to continue participating in sports [4].

Conventional treatment regimens typically include pharmacological management, immobilization, and surgery [5]. While these approaches address acute symptoms and structural damage, they often fall short in restoring optimal musculoskeletal function required for high-level athletic performance [6]. In this context, physical therapy has emerged as an essential component of interdisciplinary care for sports injuries [7]. Its scope extends beyond symptomatic relief, focusing on restoring strength, mobility, neuromuscular control, and function, while minimizing the risk of re-injury through tailored rehabilitation programs [8].

Evidence-based rehabilitation increasingly integrates a combination of physical therapy modalities, including therapeutic exercises (targeting strength and flexibility), manual therapy (hands-on mobilization of joints and soft tissues), electrotherapy (using electrical stimulation for pain relief and muscle activation), proprioceptive/balance training, and functional retraining to re-establish sport-specific skills [9,10]. By addressing biomechanical deficits, correcting muscle imbalances, and promoting proper movement patterns, physical therapists also play a preventive role in reducing future injury risk [11].

Despite accumulating evidence supporting physical therapy in managing general orthopedic conditions, there remains a need to evaluate its effectiveness specifically in sports-related injuries, particularly in local and regional contexts where epidemiological patterns and resources may differ. Understanding the outcomes of such interventions can inform clinical practice and help optimize rehabilitation strategies for athletes and active individuals.

Research objective

To evaluate the role of physical therapy in facilitating functional recovery, improving performance outcomes, and reducing recurrence rates among patients with sport-related orthopedic injuries.

## Materials and methods

Study design and setting

This descriptive observational study was conducted at the Department of Physical Therapy, Bacha Khan Medical College, Mardan, over a 12-month period from March 2023 to February 2024. The primary objective was to examine the characteristics, therapeutic approaches, and rehabilitation outcomes of patients with sports-related orthopedic injuries undergoing physical therapy.

Inclusion and exclusion criteria

Participants eligible for inclusion in the study were individuals aged 18-50 years, including those with sports-related orthopedic injuries such as sprains, strains, ligament or tendon injuries, fractures, and joint dislocations. All diagnoses were made and confirmed by board-certified orthopedic physicians based on clinical evaluation, and diagnostic imaging (X-rays, MRI, or ultrasound) was used in 148 (68.52%) cases to confirm the diagnosis. Participants were required to be actively undergoing physical therapy at the study site and to have completed at least four weeks of consistent therapy sessions prior to data collection.

Individuals were excluded if they had neurological impairments (e.g., stroke, cerebral palsy), chronic non-traumatic musculoskeletal disorders (e.g., degenerative joint disease, fibromyalgia), injuries unrelated to sports (e.g., road traffic accidents, occupational hazards), prior surgical intervention for the same injury, or inability to provide informed consent due to cognitive or communication limitations.

Sample size and sampling method

The minimum required sample size was estimated using the standard single-proportion formula as described by Lwanga and Lemeshow [12] and Pourhoseingholi et al. [13], assuming a recovery rate of 70%, a 95% confidence level, and a 7% margin of error.

$n = \frac{Z^2 . p . (1 -p){}}{d^2}$

By substituting these values into the formula, the minimum required sample size was calculated to be 165 participants to ensure adequate statistical power and precision. However, all eligible patients who met the inclusion criteria during the 12-month study period were enrolled, yielding a final sample of 216 participants. This exceeded the calculated minimum, thereby enhancing the reliability and generalizability of the study findings.

Data collection

Data were collected prospectively during routine therapy sessions using structured questionnaires and standardized clinical evaluation forms, developed in collaboration with licensed physical therapists to ensure clinical relevance (see Appendix). Trained personnel gathered information on demographic variables (age, sex, occupation, and type of sport), detailed injury characteristics (type, location, mechanism, date of occurrence, and severity as determined by the orthopedic physician), and therapy-related details (start date, duration, frequency of sessions per week, specific physical therapy modalities administered, and adherence to the prescribed rehabilitation plan). All data were entered into pre-coded electronic forms with periodic validation to ensure accuracy and completeness.

Outcome measures were assessed both at baseline and after completion of at least four weeks of physical therapy to evaluate the effectiveness of the intervention. A combination of objective clinical tools and subjective reports was employed. Pain intensity was measured using the Visual Analog Scale (VAS, 0-10) [14], while joint range of motion (ROM) was objectively quantified in degrees with a goniometer [15]. Functional performance, where applicable, was evaluated using the Lower Extremity Functional Scale (LEFS) [16]. Clinically significant improvement was defined according to validated thresholds for these standardized measures, ensuring that recovery was determined objectively rather than based solely on patient-reported perceptions.

The treatment plan for each patient was determined collaboratively by the attending orthopedic physician and the supervising licensed physical therapist, following institutional rehabilitation protocols. These protocols specified appropriate modalities and therapy progression tailored to the type and severity of injury, thereby maintaining consistency of care while allowing necessary individualization. Injury severity was classified as mild, moderate, or severe by the orthopedic physician at diagnosis, and the intensity of the sport was categorized as high-, moderate-, or variable-intensity based on established activity classifications.

Statistical analysis

Data were analyzed using SPSS Statistics, version 25 (IBM Corp., Armonk, NY, USA). Descriptive statistics (frequencies, means, standard deviations) summarized participant characteristics and outcomes. Before applying parametric tests, normality was assessed using the Shapiro-Wilk test and confirmed. Independent t-tests compared mean pre- and post-therapy values (e.g., pain scores, functional scores), and chi-square tests examined associations between categorical variables (e.g., injury type vs. recovery). Statistical significance was defined as p < 0.05.

Ethical considerations

The study received ethical approval from the Institutional Review Board (IRB) of Bacha Khan Medical College Mardan (629/DPT/BKMC; 7/12/2022). Prior to enrollment, all participants were provided with detailed information about the study objectives, procedures, and confidentiality measures. Written informed consent was obtained from each participant. All data were handled in accordance with ethical standards to ensure participant privacy and data integrity.

## Results

**Figure 1:**
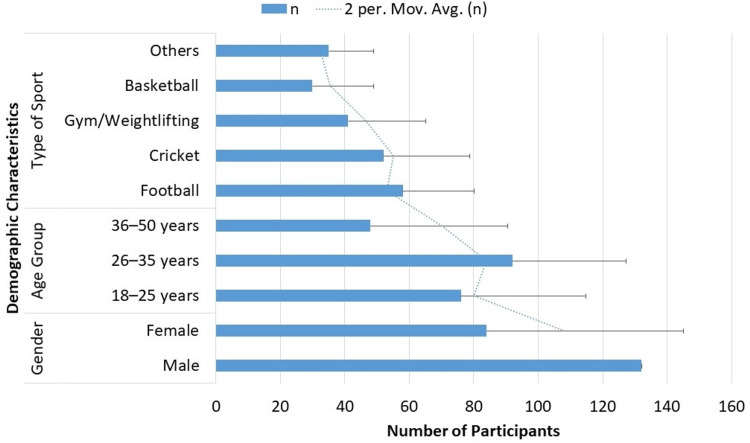
Demographic Characteristics of Participants (n = 216) The horizontal blue bar chart shows the distribution and black bars shows the percentage of study participants by gender, age group, and type of sport. The number of participants (n) is represented by the length of the bars. A 2-period moving average (dotted line) is overlaid for trend visualization.

Figure 1 summarizes the demographic distribution of the study population (n = 216) in which 61.11% (n=132) were male and 38.89% (n=84) were female. 42.59% (n=92) of the participants were between the ages of 26 and 35, followed by those between the ages of 18 and 25 (35.19%; 76) and 36 and 50 (22.22%; n=48). Football was the most popular sport, accounting for 26.85% of all sports played (n=58), closely followed by cricket (24.07%; n=52). Basketball players accounted for 13.89% (n=30), gym or weightlifting participants for 18.98% (n=41, included here as it is a structured physical activity posing orthopedic injury risks similar to other sports, n=35; 16.20%).

The most common injuries among the 216 participants were ligament sprains or tears (n = 72, 33.33%), which were followed by muscle strains (n = 49, 22.69%). Table 1 indicates that 36 individuals (16.67%) had joint dislocations, 41 (18.98%) suffered fractures, and 18 (8.33%) suffered tendon injuries. The most common injury site was the knee (n = 65, 30.09%), which was followed by the shoulder (n = 49, 22.69%), ankle (n = 44, 20.37%), elbow (n = 27, 12.50%), and hip (n = 31, 14.35%).

**Table 1:** Type and Location of Sport-Related Orthopedic Injuries

Variable	Category	Number of Patients (n;%)
Type of Injury	Ligament sprain/tear	72 (33.33)
	Muscle strain	49 (22.69)
	Joint dislocation	36 (16.67)
	Fracture	41 (18.98)
	Tendon injury	18 (8.33)
Injury Location	Knee	65 (30.09)
	Shoulder	49 (22.69)
	Ankle	44 (20.37)
	Elbow	27 (12.50)
	Hip	31 (14.35)

**Figure 2:**
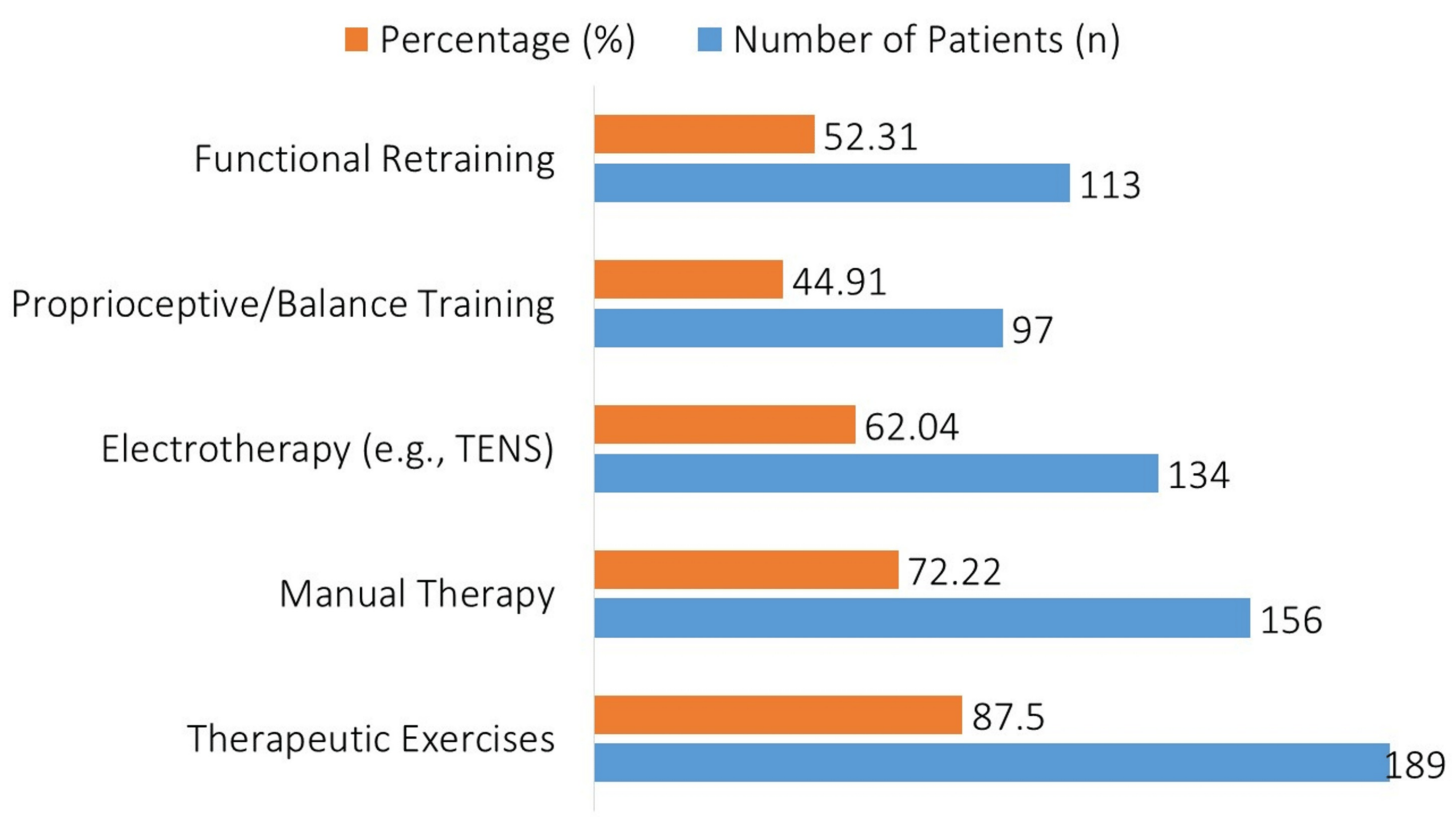
Physical Therapy Interventions Applied to Participants (n = 216). TENS – Transcutaneous Electrical Nerve Stimulation.

Figure 2 shows that of the 216 patients, 189 (87.50%) received therapeutic exercises, 156 (72.22%) received manual treatment, 134 (62.04%) received electrotherapy such as transcutaneous electrical nerve stimulation (TENS), 97 (44.91%) received proprioceptive/balance training, and 113 (52.31%) received functional retraining.

Out of the 216 patients, the majority demonstrated substantial improvement across all measured outcomes (Table 2). Specifically, 186 patients (86.11%) achieved clinically significant pain reduction on the VAS, 173 (80.09%) showed improved joint ROM, and 159 (73.61%) exhibited enhanced functional performance based on the LEFS. Additionally, 138 patients (63.89%) successfully returned to sports, while 19 (8.80%) experienced re-injury during the study period. These outcomes were determined using validated clinical thresholds for each measure, ensuring objective assessment rather than relying solely on patient-reported perceptions. Overall, functional recovery and pain relief rates exceeded 80%, highlighting the effectiveness of the rehabilitation interventions.

**Table 2:** Functional Recovery and Outcome Measures VAS – Visual Analog Scale. Recovery was defined by clinically significant improvement based on validated assessment tools.

Outcome Measure	Recovered (n)	Not Recovered (n)	Total (n)	Recovery Rate (%)
VAS Pain Reduction	186	30	216	86.11%
Improved Range of Motion	173	43	216	80.09%
Functional Performance	159	57	216	73.61%
Return to Sports	138	78	216	63.89%
Re-injury During Study	19	—	216	8.80%

Gender-based comparisons of recovery outcomes revealed no statistically significant differences in pain reduction (VAS) or ROM between males and females (Table 3). The mean VAS pain reduction scores were 7.8 ± 1.2 for males (n = 132) and 7.5 ± 1.3 for females (n = 84) (p = 0.15). Similarly, mean ROM was 85.3° ± 10.4° for males and 82.6° ± 11.1° for females (p = 0.06). However, functional performance scores were significantly higher among males (78.9 ± 9.7) compared to females (75.4 ± 10.2), with p = 0.03, indicating a modest but meaningful difference favoring males. Effect sizes and 95% confidence intervals were calculated to contextualize these findings, with the observed difference in functional performance reaching statistical and clinical significance.

**Table 3:** Comparative Analysis of Functional Recovery Outcomes by Gender (Independent t-test) *p < 0.05 indicates statistical significance. VAS – Visual Analog Scale; ROM – Range of Motion; SD – Standard Deviation.

Outcome Measure	Male (Mean ± SD)	Female (Mean ± SD)	t-value	p-value	95% CI of difference	Cohen’s d	Interpretation
VAS Pain Reduction	7.8 ± 1.2	7.5 ± 1.3	1.45	0.15	−0.11 to 0.71	0.24	Not significant
Range of Motion (ROM)	85.3° ± 10.4°	82.6° ± 11.1°	1.86	0.06	−0.08 to 5.45	0.25	Borderline
Functional Performance Score	78.9 ± 9.7	75.4 ± 10.2	2.25	0.03*	0.38 to 6.60	0.35	Statistically significant

Table 4 presents the association between injury type and functional recovery status. Recovery rates varied significantly across injury types, with higher recovery observed in patients with sprains and strains, while those with tendon injuries showed notably lower recovery. The chi-square test revealed a statistically significant association between injury type and recovery outcomes (p < 0.05), highlighting the impact of injury characteristics on rehabilitation success.

**Table 4:** Association Between Injury Type and Functional Recovery Status (Chi-square Test). Chi-square test: χ². Significant association between type of injury and functional recovery (p < 0.05).

Injury Type	Recovered (n, %)	Not Recovered (n, %)	Total (n)	χ² (df)	P-value
Ligament sprain/tear	58 (80.56%)	14 (19.44%)	72	18.42 (4)	0.001
Muscle strain	42 (85.71%)	7 (14.29%)	49		
Joint dislocation	24 (66.67%)	12 (33.33%)	36		
Fracture	29 (70.73%)	12 (29.27%)	41		
Tendon injury	6 (33.33%)	12 (66.67%)	18		
Total	159 (73.61%)	57 (26.39%)	216	-	-

## Discussion

This research assessed the effectiveness of physical therapy in helping 216 participants - mostly male (61.11%, n=132) and mostly between the ages of 26 and 35 (42.59%, n=92) - recover from orthopaedic injuries sustained during sports. These demographic patterns are consistent with other research that found that young to middle-aged persons are more likely to sustain sports injuries because they engage in greater physical activity [17,18]. The gender distribution also supports research showing that men are more likely to participate in high-risk sports and get injuries as a result [19].

The most frequent injury type in this group was ligament sprains or tears (33.33%, n=72), which were followed by tendon injuries (8.33%, n=18), fractures (18.98%, n=41), muscle strains (22.69%, n=49), and joint dislocations (16.67%, n=36). The most often impacted joint (30.09%, n=65) was the knee, which is in line with previous research showing that knee injuries account for the majority of sport-related trauma, especially in basketball and football [20]. This demonstrates why specific rehabilitation regimens emphasizing knee stability and proprioception are necessary.

The majority of patients received therapeutic exercises (87.50%, n=189), which were the most common intervention. Manual therapy (72.22%, n=156), electrotherapy (62.04%, n=134), functional retraining (52.31%, n=113), and proprioceptive training (44.91%, n=97) were the next most common interventions. These results are in line with other studies that highlight the critical role manual treatment and therapeutic activities play in improving functional recovery after an accident [21].

Significant improvements were seen in functional outcomes: 73.61% (n=159) increased functional performance, 86.01% (n=186) reduced discomfort (VAS), and 80.09% (n=173) improved range of motion. Remarkably, 63.89% (n=138) of participants were able to resume their athletic activities, and the re-injury rate was comparatively low at 8.80% (n=19). These outcomes are in line with earlier research that found that organized physical therapy regimens led to pain alleviation and functional restoration rates of over 75% [22]. The success of tailored rehabilitation in promoting a safe transition back into athletic activity is further shown by the return-to-sport rate.

Males scored considerably better on functional performance (p=0.03), but there was no significant difference in pain reduction (p=0.15) or range of motion improvement (p=0.06) according to gender-based analysis. Previous research has shown similar gender disparities in recovery results, which have been ascribed to differences in muscle strength, endurance, and biomechanical variables [23,24]. The finding raises the possibility that rehabilitation methods need to be modified to account for gender. These results support the critical role that comprehensive physical therapy plays in treating orthopedic injuries connected to sports, highlighting a multimodal strategy that targets pain, strength, mobility, and neuromuscular function to maximize recovery results.

Study strengths and limitations

This study's strength is a relatively large and diverse sample of 216 physically active individuals with a broad spectrum of sport-related orthopedic injuries, enhancing the generalizability of findings within the 18-50 age group. The use of validated, standardized clinical tools such as the VAS for pain, goniometric ROM measurement, and the LEFS for functional performance allowed for objective and reproducible assessment of recovery outcomes. The inclusion of multiple physical therapy modalities reflects real-world clinical practice and adds practical relevance to the findings.

To reduce potential bias, trained professionals collected data during therapy sessions using structured questionnaires and pre-coded electronic forms, with periodic validation of entries to ensure accuracy and completeness. Injury severity was documented at baseline and sports intensity was classified by type of sport to account for these potentially confounding factors, although detailed subgroup analysis was beyond the study scope.

Despite these strengths, the descriptive observational design limits causal inference, and the convenience sampling approach may introduce selection bias. The absence of a control group and long-term follow-up are additional limitations. Moreover, psychosocial factors such as family dynamics and social support, which could influence adherence and recovery, were not assessed. Future studies should incorporate these variables, include long-term follow-up, and consider controlled or randomized designs to strengthen causal conclusions.

## Conclusions

The results of this research demonstrate how important physical therapy is in helping individuals with orthopedic injuries connected to sports achieve meaningful functional recovery. Most participants reported significant pain alleviation, increased mobility, and better functional performance; a significant percentage of them were able to resume their sporting activities. The low risk of re-injury provides more evidence of the efficacy of personalized, organized rehabilitation regimens. These findings support physical therapy's importance in treating sports injuries and encouraging a safe and effective return to athletic activity.

## Appendices

**Table 5:** Standardized Data Collection Questionnaire VAS – Visual Analog Scale; ROM – Range of Motion

Section	Question	Response Type	Response
Demographics	Age	(Years)	
	Sex	Male / Female / Other	
	Occupation	Open-ended	
	Sport Played	Open-ended	
	Level of Participation	Recreational / Competitive	
Injury Details	Injury Type	Sprain / Strain / Fracture / Ligament Injury / Tendon Injury	
	Injury Site	Upper limb / Lower limb / Spine / Other (specify)	
	Side Affected	Left / Right / Bilateral	
	Injury Date	Date	
	Mechanism of Injury	Sudden trauma / Overuse / Fall / Collision / Other	
Physical Therapy Details	Therapy Start Date	Date	
	Duration of Therapy	Weeks	
	Sessions per Week		
	Therapy Modalities Used	Manual therapy / Electrotherapy / Exercise / Combination	
	Adherence to Therapy	Regular / Irregular	
Outcome Measures	Pain Level (VAS) Before Therapy	Scale 0–10	
	Pain Level (VAS) After Therapy	Scale 0–10	
	ROM Before Therapy	Degrees (Specify Joint)	
	ROM After Therapy	Degrees (Specify Joint)	
	Functional Score Used	LEFS / SPADI / DASH / Other	
	Functional Score Before Therapy		
	Functional Score After Therapy		
Patient Feedback	Satisfaction with Recovery	Very Satisfied / Satisfied / Neutral / Dissatisfied	
	Willingness to Recommend Therapy	Yes / No	
